# High-resolution an**a**lysis of DNA copy number alterations in patients with isolated sporadic keratoconus

**Published:** 2011-03-30

**Authors:** Khaled K. Abu-Amero, Ali M. Hellani, Sameer M. Al Mansouri, Hatem Kalantan, Abdulrahman M. Al-Muammar

**Affiliations:** 1 Ophthalmic Genetics Laboratory, Department of Ophthalmology, College of Medicine, King Saud University, Riyadh, Saudi Arabia; 2 Saad Specialist Hospital, Al-Khobar, Saudi Arabia; 3 Anterior Segment Unit, Department of Ophthalmology, College of Medicine, King Saud University, Riyadh, Saudi Arabia

## Abstract

**Purpose:**

To determine whether patients with sporadic, non-familial keratoconus and no pathogenic mutations in the visual system homeobox 1 (*VSX1*) gene have evidence of chromosomal copy number alterations.

**Methods:**

Twenty Saudi Arabian patients with isolated keratoconus, no family history of the disease and no mutations in *VSX1* were recruited. Additionally, 10 ethnically-matched healthy controls were also recruited for this study. We screened patients for chromosomal copy number aberrations using the Agilent Human Genome CGH 244A Oligo Microarray Chip.

**Results:**

None of the keratoconus patients screened had evidence of chromosomal copy number alterations when compared to normal ethnically matched controls.

**Conclusions:**

Chromosomal deletions and/or duplications were not detected in any of the patients tested here. Other chromosomal imbalances such as translocations, inversions, and some ploidies cannot be detected by current array CGH technology and other nuclear genetic or epigenetic factors cannot be excluded as a possible contributing factor to keratoconus pathogenesis.

## Introduction

Keratoconus (KTCN; OMIM 148300) is a non-inflammatory thinning and anterior protrusion of the cornea that results in steepening and distortion of the cornea, altered refractive powers, and altered visual acuity. In more advanced cases, corneal scarring from corneal edema, and decompensation further reduces visual acuity. Symptoms are highly variable and depend on the stage of progression of the disorder [1]. The incidence of Keratoconus ranges between 1/500 to 1/2000 individuals throughout the world [2]. The disease occurs with no ethnic or gender preponderance and causes significant visual impairment [2-4]. Most cases of keratoconus are sporadic but a minority (5%–10%) has positive family history [3,5]. In such cases both autosomal recessive and dominant patterns of inheritance have been reported [6-9]. There are several chromosomal loci and genes reported to be associated with keratoconus [3,9]. However, some were eventually excluded [3,10], while others no confirmed association with the disease have been established [11,12]. This is not the case for the visual system homeobox 1 (*VSX1*) gene where mutations associated with keratoconus cases have been found in different studies [13-16]. Other studies did not report *VSX1* mutations in diverse population cohorts of keratoconus patients [17,18], including Saudi keratoconus patients [19]. This indicates that keratoconus is a complex condition of multi-factorial etiology and that mutations in *VSX1* are not responsible for all cases of keratoconus.

Keratoconus can be divided into three broad categories: i) keratoconus associated with rare genetic disorders (such as Down syndrome, nail-patella syndrome, neurofibromatosis, etc); ii) keratoconus in the setting of commonly reported associations (contact lens wear, eye rubbing, atopy, Leber congenital amaurosis, mitral valve prolapsed and positive family history) and iii) isolated keratoconus with no associations. To our knowledge, no study has investigated chromosomal copy number variations in patients with any of the three types of keratoconus mentioned above.

Here we investigate the possible presence of chromosomal copy number changes in patients with isolated non-familial keratoconus using high resolution array comparative genomic hybridization (array CGH) technology. To our knowledge, this investigation was not performed anywhere else previously.

## Methods

### Patients and controls

Patients were selected from the anterior segment clinic at King Abdulaziz University Hospital, King Saud University in Riyadh, Saudi Arabia after full ophthalmological examination by anterior segment specialists (A.A. and H.K.). Patients were diagnosed with keratoconus if the Schimpff-flow based elevation map showed posterior corneal elevation within the central 5 mm ≥+20 µm, inferior-superior dioptric asymmetry (I-S value) >1.2 Diopter (D) and the steepest keratometrey >47 D [20-22]. We have chosen these parameters to exclude cases that are keratoconus suspects and to confine our study group to only cases with definite keratoconus. All our patients were examined by a specialist and established to be free of any genetic disorder commonly associated with keratoconus [3,21]. Patients were labeled as sporadic after examining the immediate family members and identifying the patient as isolated case of keratoconus. Exclusion criteria was post-LASIK (laser-assisted in situ keratomileusis), ectasia, or has a family history of keratoconus or more than one individual from the same immediate family were affected. All study subjects were self identified of Saudi Arabian ethnicity. Family names were all present in the database of Arab families of Saudi Arabian origin. All keratoconus cases secondary to causes like trauma, surgery, Ehlers Danlos syndrome, osteogenesis imperfecta and pellucid marginal degeneration were excluded from the study.

The controls were recruited from the general ophthalmology clinic that had no ocular disease(s) or previous ophthalmic surgeries. Their slit lamp exam showed clear cornea and their Schimpff-flow based elevation map was within normal limit. This research adhered to the tenets of the Declaration of Helsinki, and all patients and controls signed an informed consent approved by the institutional review board.

### Array CGH technique

Blood was collected in ACD tubes and DNA extracted using the Qiagen Autopure LS instrument (Qiagen, Valencia, CA) following the manufacturer recommended procedure. To detect chromosomal rearrangements, 2 μg of keratoconus patient genomic DNA was competitively hybridized with 2 μg of ethnicity matched control DNA (as a reference sample) on an Agilent Human Genome CGH 244A Oligo Microarray Kit (Agilent Technologies, Inc., Santa Clara, CA), which has an average probe spacing across the human genome of 6.4 Kb. Briefly, 3 μg of DNA from keratoconus patients and controls was digested using 50 units of Alu1 (Roche, Mannheim, Germany) and 50 units of Rsa1 (Roche) restriction enzymes in a 100 μl volume with 10 μl 10× Promega Buffer C. Digestions were performed for 2 h at 37 °C. Digested samples were purified using QIAprep Spin Miniprep columns (Qiagen) and eluted according to the manufacturer's instructions. Samples were then analyzed using the Agilent 2100 Bioanalyzer with the DNA 7500 LabChip Kit and DNA 7500 Software Script (Agilent) as per the manufacturer’s instructions. Alu1/Rsa1 digested DNA samples were labeled using the BioPrime Array CGH Labeling Kit (Invitrogen, Carlsbad, CA) according to the manufacturer's protocol. Keratoconus patient and control DNA samples were systematically labeled with Alexa Fluors 555 and 647, respectively.

Labeled products of each sample and control DNA were purified using QIAprep Spin Miniprep columns (Qiagen), mixed together, and checked on the Agilent 2100 Bioanalyzer (Agilent) to evaluate the Alexa Fluors 555 integration into the DNA samples. The following hybridization blocking reagents were added to the purified Alexa Fluors 555 and 647 labeled samples: 50 μg Cot-1 DNA (Invitrogen) and 50 μl 10× control targets (Agilent). The volume was brought to 250 μl with ddH_2_O, and 250 μl 2× hybridization buffer (Agilent) was added. The hybridization mixture was then denatured at 100 °C for 3 min in a water bath. Samples were immediately transferred to a 37 °C water bath for 30 min to allow pre-annealing of the blocking agents to the labeled sample. Samples were centrifuged for 5 min at 16,000× g and immediately applied to the Agilent Human Genome CGH 244A Oligo Microarray Kit (Agilent) as per the manufacturer's recommendations. Hybridizations were performed at 65 °C for 42 h.

Microarrays were disassembled in Agilent wash buffer-1 at room temperature (RT), transferred to a slide holder, and incubated for 5 min with stirring in the Agilent wash buffer-1 at RT. The second washing step was performed for 1 min in wash buffer-2 at 37 °C. The third and fourth washing steps were done with acetonitrile (Fisher Scientific, Fair Lawn, NJ) and stabilization solutions (Agilent) for 1.5 min at RT, respectively. Microarray slides were immediately scanned in the Agilent DNA Microarray Scanner using the default settings.

Data analysis was performed using Agilent Feature Extraction 9.1 and CGH Analytics 3.4 (Agilent). Log^2^ expression ratios were computed and normalized using CGH Analytics 3.4 software. Putative chromosome copy number changes were defined by intervals of 3 or more adjacent probes with log^2^ ratios suggestive of a deletion or duplication when compared with the log^2^ ratios of adjacent probes. The quality-weighted interval score algorithm (ADM2) was used to compute and assist in the identification of aberrations for a given sample. A more detailed protocol of the array CGH protocol using the Agilent platform was previously reported [23].

**Figure 1:**
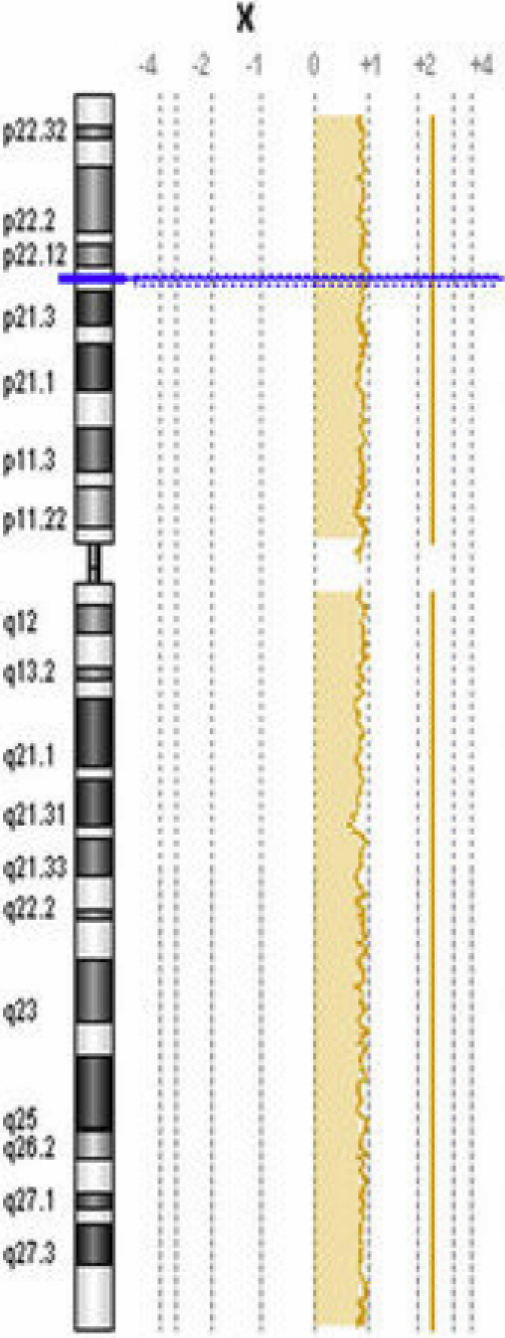
Array CGH result for internal control. As an internal quality control for the array CGH procedure, opposite sex control DNA was hybridized against keratoconus DNA (ratio of +1 with regard to chromosome X for XX keratoconus and XY control).

As an internal quality control measure, DNA from Saudi keratoconus-patients were mixed with DNA from controls (free of keratoconus) of the same and the opposite sex and co-hybridized to the 244K Agilent-chip (Figure 1).

## Results

Clinical characteristics of the 20 keratoconus patients included in this study are detailed in Table 1. None of our patients had a family history of keratoconus and all were the only affected members of their respective family. Patient sex (9 males and 11 females) and mean age of 25.4 (SD 7.3) and ethnicity matched controls (5 males and 5 females) with a mean age of 58 (SD 15) were clinically evaluated as detailed in the methods. Patient charts were reviewed carefully and all patients were defined as isolated keratoconus cases with no association with genetic or systemic disorder. The entire coding region of *VSX1* was screened and no pathogenic mutation(s), either novel or previously reported were found [19].

**Table 1:** Clinical phenotypes of keratoconus patients.

**Patient demographics**			**Uncorrected visual acuity in Snellen's chart**		**Munsen sign**		**Vogt's striae**		**Hydrops**		**Scarring**		**Average keratometry in VKG (in Diopters)**		**Optical pachymetry (mm)**	
**ID**	**Age**	**Sex**	**OD**	**OS**	**OD**	**OS**	**OD**	**OS**	**OD**	**OS**	**OD**	**OS**	**OD**	**OS**	**OD**	**OS**
1	20	M	20/100	20/200	+	+	+	+	+	+	-	+	51.9	64.7	525	502
2	18	M	CF	20/80	-	-	+	+	+	+	+	-	58	56	300	346
3	17	M	CF	20/100	+	-	+	+	-	-	-	-	60	56.6	455	509
4	35	F	20/20	20/60	-	-	-	+	-	+	-	+	43.1	44.6	584	554
5	30	M	20/200	CF	-	-	-	-	-	-	-	-	49.1	68.7	434	349
6	36	M	20/100	20/100	-	-	-	-	+	+	-	-	48.4	50.4	459	439
7	16	M	CF	CF	+	+	+	+	+	+	-	+	54.4	55	241	268
8	24	M	CF	20/80	+	+	+	+	+	+	+	+	53	65.7	302	397
9	25	F	CF	CF	+	+	+	+	-	-	+	-	67.3	70.7	236	216
10	25	M	20/20	CF	-	+	-	+	-	+	-	-	43.1	50.9	419	407
11	32	F	20/40	20/40	+	+	+	+	+	+	-	-	43.5	51.7	442	398
12	20	F	20/25	20/100	-	-	-	+	-	+	-	-	42.7	56.2	509	456
13	32	F	CF	20/200	-	-	-	-	+	+	-	-	56.3	52.6	429	471
14	17	F	CF	20/25	+	+	+	-	+	+	+	+	69.3	45.5	284	522
15	24	F	20/200	CF	-	+	-	+	+	+	-	+	49.8	62.2	492	218
16	39	F	20/200	20/200	-	-	-	-	+	+	-	-	49.2	53.1	458	419
17	25	M	20/40	CF	+	+	+	+	+	+	-	+	42.8	43.7	482	511
18	17	F	20/60	20/100	+	+	-	+	+	+	-	-	56.4	58.2	411	414
19	22	F	20/100	20/100	-	-	-	-	+	+	-	-	46.4	44.5	425	416
20	35	F	CF	CF	-	-	-	-	-	-	-	-	43.6	63.1	482	371

Key: M=Male; F=Female; OD=Right eye; OS=Left eye; +=Positive; -=Negative; VKG=Videokeratography; AD=Autosomal dominant; AR=Autosomal recessive; SP=Sporadic; ND=not determined due to difficulty in predicting the mode of inheritance from available family pedigree. All patients listed in this table were sporadic cases of keratoconus as determined by examining the family pedigree carefully and taking detailed family history up to 2–3 generations.

**Figure 2:**
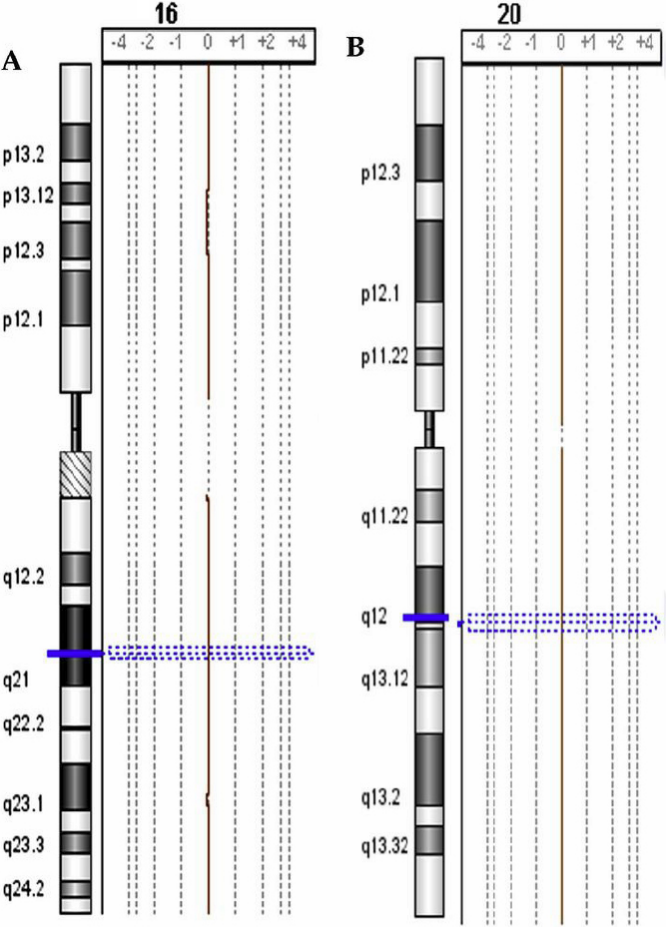
Array CGH results for keratoconus patients versus controls. Chromosomes shown were chosen randomly as representative of all chromosomes and in all Keratoconus patients tested. **A**: Chromosome 16 and **B**: Chromosome 20. When control DNA was hybridized against patient’s DNA, a signal ratio of zero (0) was obtained, indicating the absence of chromosomal copy number alterations.

We proceeded with screening the patients for gross chromosomal abnormalities. The signal ratio of each patient compared to a simultaneously tested control (patient-cy3/control-cy5) documented the absence of chromosomal copy number variations in any patient. Representative images of array CGH results are shown in Figure 2.

## Discussion

The 20 patients reported here met rigorous clinical criteria for clinically defined keratoconus as detailed in methods. They were all sporadic cases and were identified as Saudi Arabians. We also recruited 10 healthy controls (free of keratoconus). Mutations in *VSXI* have been identified in association with keratoconus [13-15], but in our cohort, we did not detect any mutations in this gene [19]. This may not be surprising as the role of *VSXI* in keratoconus is still ambiguous and many studies in various populations found no mutations in this gene in their respective populations [12,17,18,24]. Human *VSXI* is a member of the CVC domain containing paired-like class of homeo-proteins. *VSXI* expression in humans is detected in embryonic craniofacial, adult retinal, and adult corneal tissues. Previous studies have shown that the pathogenesis of keratoconus is very complex and several gene- and gene-environmental interactions play a critical role in disease prognosis.

Since its introduction as a technique to detect genomic imbalances, array-based comparative genomic hybridization (array CGH) has revolutionized our understanding of the structure of the human genome and greatly improved the study of tumors and is rapidly becoming a new standard method for clinical cytogenetics [25]. High resolution array CGH used here provides quantitative information about the level of chromosome gain or loss, such as regions with high-level amplification or high-magnitude deletion, and will recognize a chromosomal duplication or deletion of a size of about ≥6 Kb. This technique did not detect any chromosomal copy number variations of this size in keratoconus patients or controls. These results indicate that it is very unlikely that chromosomal deletions or duplications are universally responsible for isolated cases of keratoconus. Because of the relatively small sample size, it remains possible that chromosomal aberrations might be present in a portion of patients with isolated keratoconus. More patients from multiple centers and various ethnicities would need to be examined to make a general statement about the absolute absence of chromosomal copy number variations in the setting of isolated keratoconus. No comment can be made about other chromosomal imbalances such as translocations, inversions, and some ploidies because these cannot be detected by the current array CGH technology.

In summary, we used high resolution array CGH to evaluate a group of patients with isolated Keratoconus and found no evidence of chromosomal copy number variations. Therefore, currently neither pathogenic mutations nor chromosomal deletions/duplications provide a complete explanation for isolated cases of Keratoconus in our patients. Although unrecognized genetic or epigenetic factors may play a role in keratoconus-pathogenesis.
